# From Health Campaign to Interpersonal Communication: Does Traditional Diet Culture Hinder the Communication of the Chinese *Gongkuai* Campaign?

**DOI:** 10.3390/ijerph19169992

**Published:** 2022-08-13

**Authors:** Jing Yan, Jing Ji, Lan Gao

**Affiliations:** 1 School of Health Service Management, Anhui Medical University, No. 81, Meishan Road, Hefei 230032, China; 2 School of Management, University of Science and Technology of China, No. 96, Jinzhai Road, Hefei 230026, China

**Keywords:** health campaign, interpersonal communication, traditional diet culture, health risk perception, *Gongkuai* campaign

## Abstract

Interpersonal communication is beneficial in promoting individuals’ tendency to accept health-campaign-targeted behavior. Based on the protective action decision model, this study investigated the key factors underlying individual’s interpersonal communication on the *Gongkuai* campaign, which was carried out during Coronavirus disease 2019 (COVID-19). The main goal of the *Gongkuai* campaign was to change traditional communal eating habits and reduce public health risks. An online questionnaire survey involving 618 respondents was conducted in China after the 2020 *Gongkuai* campaign propagated, and the data were analyzed by using the structural equation modeling technique. The results indicated that health campaign exposure is a critical determinant of perceived campaign-related knowledge and health risk perception, which are significant predictors of interpersonal communication. Meanwhile, campaign-related knowledge can elicit risk perception. Furthermore, campaign exposure influenced interpersonal communication in ways that traditional diet culture did not predict. Risk perception was also unaffected by traditional diet culture. It is worth noting that individuals’ agreement with traditional diet culture does not hinder health campaign-generated interpersonal communication in the context of public health crisis. Based on the findings, theoretical and policy implications for motivating interpersonal communication were discussed, and research limitations were pointed out.

## 1. Introduction

The outbreak of COVID-19 in China in 2019 sparked anxiety worldwide, causing significant economic and psychological harm [1]. Fortunately, the spread of the virus can be slowed if the public takes appropriate precautions, such as wearing a face mask and not sharing food and drinks. Following the coronavirus outbreak, the Chinese government is pushing for a “dining table revolution” (a national campaign to persuade diners to use designated serving chopsticks known as public chopsticks, short for “*GongkKuai* Campaign”) to change long-held traditions of communal eating habits, in which diners take from shared plates with their own chopsticks (https://uk.news.yahoo.com/chinas-dining-table-revolution-takes-104024594.html, accessed on 1 May 2020). Celebrities, public health experts, and propaganda officials have disseminated health information about the use of serving chopsticks through various information channels such as television, radio, newspaper and social media, etc. As a result, the *Gongkuai* campaign has become a hot sociopolitical issue that received extensive media coverage.

The use of serving chopsticks during meals has been emphasized in literature as an important factor in decreasing the transmission of bacteria among family members [2,3]. The *GongKuai* campaign, on the other hand, has raised concerns about whether the pandemic will change the country’s time-honored communal eating tradition that has been a dominant part of social life (http://www.globaltimes.cn/content/1181261.shtml, accessed on 1 March 2020). A similar campaign was launched after the 2003 SARS epidemic, but it was not propagated effectively [4]. Culture has long been recognized as having a significant impact on shaping an individual’s health behaviors [5]. Prior research explained why using serving chopsticks is a difficult feat to accomplish, given that the shared use of chopsticks has been a part of Chinese culture for many centuries [6,7]. 

However, the data on this topic are limited. A previous study showed that the lack of behavior change could result from the failure to adapt the intervention to the culture [8]. This fact was experimentally proven by [9] that social culture is an important barrier to the use of serving chopsticks. In health information research, [10] suggested that cultural values determine how immigrants seek health information in the United States. However, it has not been a priority to explore the role of cultural values on interpersonal communication in the context of large-scale health campaigns. Considering that interpersonal communication is critical in disseminating health information from mass media to the public, various studies have suggested that large-scale health campaigns can be extended through interpersonal communication [11]. Research further proposed that conversations stimulated by health campaign messages are more effective at delivering campaign-directed outcomes than simple exposure to the mass media messages [12]. That is to say, campaign-generated interpersonal communication is significant in bringing about desired health outcomes or health behavior change. As a result, it appears that whether and how people communicate information about the *Gongkuai* campaign is a critical factor in improving the public’s willingness to use serving chopsticks.

To fill the research gap, current research aims to examine the effect of *Gongkuai* campaign exposure and traditional diet culture on campaign-generated interpersonal communication. To achieve this goal, the Protective Action Decision Model (PADM) was adopted as the basic theoretical framework, derived initially from the literature on public protective actions in response to impending disasters (Lindell & Perry, 2012) [13]. Later, this theory was extended to explain people’s long-term risk adjustment. According to PADM, socially transmitted warnings initiate a series of preliminary decision-making processes, generating core perceptions of the external threat and alternative protective measures. In the following sections, firstly, we introduced PADM and then proposed the research hypotheses; later, the methodology of data collection was described before conducting the analysis. Finally, we discussed the results and implications and pointed out the limitations and future research directions.

## 2. Theory and Hypotheses

### 2.1. Interpersonal Communication and Protective Action Decision Model (PADM)

Since most health communication campaigns are based on media outlets such as television and social networking sites, expanding health information through interpersonal communication with the public is the first and important step in achieving public health objectives. Therefore, interpersonal communication can be seen as rational and protective behavior [14]. In contrast to other health campaigns (e.g., blood donation), which focus on personal health behavior, the *Gongkuai* campaign can only be successful if a wide range of people participate. People who effectively communicate health information are more receptive to suggestions from health campaigns and are more likely to change their unhealthy habits [15,16]. Thus, the communicative action of sharing and discussing health messages can stimulate outcomes such as obtaining personal goals [11]. Intuitively, *Gongkuai*-related conversations can be seen as a goal-oriented and protective action in which individuals discuss the health campaign with others (e.g., families, friends, and coworkers) to raise awareness regarding the importance of using serving chopsticks. 

**Figure 1:**
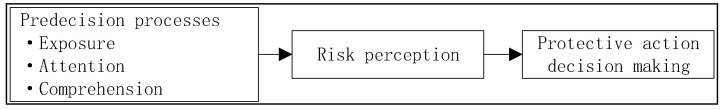
Psychological Processes of PADM.

PADM is a crucial model for investigating people’s protective actions [13]. According to this multistage model, psychological processes (Figure 1) are the main stages that illustrate people’s response to environmental threats. Moreover, in the psychological processes of PADM, risk perception is a major determinant of protective measures relating to people’s expectations of the personal impact of an extreme public event. These personal impacts include death, injury, health, and property damage [17]. PADM proposed that in the context of public emergencies, people’s risk perception was influenced by a combination of event-related information from external sources and past knowledge of the individual. People will decide to take protective action if a risk is perceived to exist. In the past decades, several scholars have used PADM to illustrate people’s protective action in response to social risks, such as water contamination emergencies [18], influenza outbreaks [19], and vaccine scandals [20]. This theory allows us to identify core variables (health campaign-related knowledge, information exposure, risk perception, etc.) to predict how *Gongkuai*-related information is shared and diffused. Thus, we tentatively adopted PADM to explain how information from external sources, risk perception, and campaign-related knowledge influenced information-sharing behavior in the context of the COVID-19 outbreak.

### 2.2. Campaign Exposure, Campaign-Related Knowledge, and Risk Perception

Health campaign exposure refers to the frequency and duration of information delivered via mass and social media about the benefits of using serving chopsticks [21]. Additionally, campaign-related knowledge refers to an individual’s tendency to use serving chopsticks as well as their familiarity with the *Gongkuai* campaign. Individuals’ health knowledge increases because of media exposure which subsequently triggers positive health behavior [22,23]. For example, residents’ perceptions about the benefits of waste separation are based on adequate exposure to information through a waste separation campaign [24]. Furthermore, [22] demonstrated that vaccine-related health campaigns are significant reasons for increasing awareness in parents regarding the vaccination of their children. However, a prior study suggested that exposure to a campaign does not improve people’s recognition of campaign-related messages [25]. As a result, further studies are necessary to see if exposure to the *Gongkuai* campaign is an essential predictor of individuals’ health knowledge about using serving chopsticks.

Furthermore, many health campaigns aim to raise public awareness and risk perceptions of unhealthy behaviors to render the behavior of interest more or less appealing [26]. Health risk perception is defined as an individual’s response to the possibility of developing a disease or illness [27]. Repeated media exposure to a health crisis can lead to increased worry and vulnerability [28]. Seo and Matsaganis [29] found that exposure to media reports increases an individual’s risk perceptions regarding breast cancer. Other research scholars [30] found that media exposure positively influences adolescents’ smoking risk perception. Based on the evidence presented above, it appears that exposure to the *Gongkuai* campaign-related information will enhance risk perceptions of not using serving chopsticks. 

In addition, the knowledge people have about health behavior is related to their risk perception. For example, Perettiwatel et al. [31] showed that highly educated people with greater knowledge of certain health behavior are more likely to perceive the risks associated with noncompliance. Similarly, other studies found that people who know more about diabetes are more likely to be aware of its negative consequences [32]. This argument is consistent with previous research showing that the provision of knowledge about the causes and consequences of unhealthy behavior (such as smoking) is significantly correlated with health risk concerns [33].

Based on the viewpoints mentioned above, the following hypotheses can be proposed:

**Hypothesis** **1:** *Higher levels of Gongkuai campaign exposure will be associated with higher levels of individuals’ knowledge of using serving chopsticks*.

**Hypothesis** **2:** *Higher levels of Gongkuai campaign exposure will be positively associated with level of individuals’ health risk perception of noncompliance with using serving chopsticks*.

**Hypothesis** **3:** *Higher levels of health knowledge of using serving chopsticks will be positively associated with levels of individuals’ risk perception of not using serving chopsticks*.

### 2.3. The Role of Traditional Diet Culture

Culture is widely accepted as a factor that plays an essential role in public health communication programs and interventions [5,34]. Traditional diet culture refers to engagement, respect, and acceptance of customs and norms of traditional diet values and practices [35]. Additionally, traditional diet culture can affect an individual’s food intake and physical behavior [36,37]. The most popular reflection of traditional culture in most Chinese dining situations is communally shared dishes [7,38]. Communal eating habit is an integral part of Chinese traditional dietary culture. Using serving chopsticks and spoons to prevent COVID-19 infection is encouraged as a part of the *Gongkuai* campaign, even though it goes against Chinese tradition. 

Most studies investigated how traditional diet culture influences health behaviors [39,40]. Zhang et al. [9] found that more than half of the participants did not use serving chopsticks despite being aware of COVID-19 infection risk because such behaviors contradict their traditional diet culture. Similarly, Arroyo and Harwood [41] observed that women are more likely to experience physical problems when culturally idealized body type norms are associated with slenderness and toned physique. That is to say, culture shapes health-related values and beliefs [5]. In this study, it can be speculated that health risk perception is negatively influenced by an individual’s awareness of traditional dining culture. A related hypothesis can be proposed as follows:

**Hypothesis** **4:** *Higher levels of health risk perception of not using serving chopsticks will be negatively associated with level of agreement with traditional diet culture*.

Given that open discussion of specific health campaign is inappropriate according to existing social norms in India, Frank et al. [42] found that risk perception of HIV through sexual transmission is less likely to form interpersonal communication of the condom normalization campaign. The research on immigrants’ acculturation also showed that people with a strong desire to maintain their original health culture’s traditions and values are more likely to seek and adopt health information from websites hosted in their original culture [15]. Individuals who have a strong attachment to their native culture, on the other hand, may be less likely to engage in health information behaviors such as seeking new healthcare knowledge or discussing a new healthcare culture with their friends [10,43]. Conversely, new health information is more likely to be passed on and adopted if people feel that it is compatible with their existing norms [15]. Due to the incompatibility of Chinese traditional dining culture and using *Gongkuai*, it can be hypothesized that:

**Hypothesis** **5:** *Higher levels of agreement with traditional diet culture of dining will be negatively associated with campaign-generated interpersonal communication*.

### 2.4. Motivation for Campaign-Generated Interpersonal Communication

The risk perception in any specific context is an antecedent of information behaviors [13]. Emotions such as fear and sadness may particularly engender social communication [44]. Feelings of worry and anxiety about a risky situation may promote one’s motivation to use and share more gathered health information [16,45]. For example, women with more risk perception associated with having unsafe sex in the context of exposure to safer sex media campaigns are more inclined to discuss this theme with their friends or significant others [46]. Moreover, under some circumstances, people who feel uncertain about themselves generally consider simple talk within their social networks as a useful and predominant means for self-verification [29]. Based on the above views, it can be concluded that perception of health risks will increase an individual’s willingness to talk and discuss specific health campaigns. Hence, it is hypothesized that:

**Hypothesis** **6:** *Higher levels of health risk perception will be positively associated with interpersonal communication about the Gongkuai campaign*.

Individuals may be unable to engage in related information behaviors due to a lack of knowledge about them [24]. Jepsen [47] found that people with a high level of knowledge are more confident in determining what information they require and obtaining it effectively. These people are more likely to participate in health communication. Similarly, according to [48], existing health knowledge can predict chronic patients’ willingness to share information. Kim et al. [49] proposed that the objective component of prior knowledge can increase motivation to share information with others. On the other hand, people with a low level of health knowledge are less likely to engage in health information behaviors [50].

In addition, there is much empirical evidence suggesting that mass media health campaigns can promote interpersonal communication [14,51]. Like in early research [52], it was found that women who were more exposed to AIDS-related information through mass media were significantly more likely to discuss the disease within their social networks. Additionally, if individuals are fully informed about health campaign-targeted behaviors, they will be more likely to conduct conversations about the health campaign [53,54]. As a result, exposure to media health campaigns may encourage people to talk about specific health issues. Taking these perspectives into account, related hypotheses are stated as follows:

**Hypothesis** **7:** *Higher levels of health knowledge will be positively associated with interpersonal communication about the Gongkuai campaign*.

**Hypothesis** **8:** *Higher levels of health campaign exposure will be positively associated with interpersonal communication about the Gongkuai campaign*.

**Figure 2:**
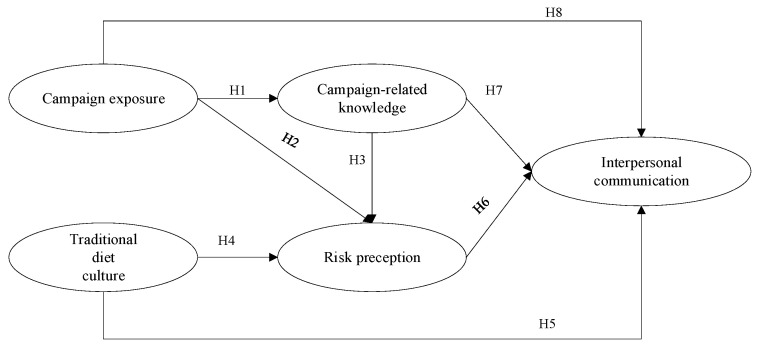
Research framework of interpersonal communication about *Gongkuai* campaign.

Based on the above analysis, the research framework and model are proposed in Figure 2.

## 3. Research Method

### 3.1. Data Collection and Samples

Since the post-lockdown period of the COVID-19 pandemic in China, the *Gongkuai* campaign has aimed to raise awareness, inform about, and encourage the use of utensils. This research was conducted in the form of a questionnaire survey after the *Gongkuai* campaign, which was officially propagated across the country in March 2020. The questionnaire was divided into four sections based on a review of previous studies. The first section briefly explained the study’s purpose and expressed gratitude for the respondent’s participation. The second part provided an overview of the *Gongkuai* campaign’s progress to refresh respondents’ memories of the campaign. The third part contained lists of items designed to identify scales of constructs. The final part included questions regarding demographics and eating habits.

Due to the prevailing COVID-19 pandemic, the online survey was conducted through a professional survey platform—Wenjuanxing (www.sojump.com (accessed on 2 April 2020), a website similar to SurveyMonkey), which is a widely accepted online questionnaire survey platform in china for data collection and has more than 28.7 million registered members [55]. A sample of 1000 people was randomly selected as potential participants from the registered members of Wenjuanxing. The survey link and a brief introduction were then distributed to these 1000 potential participants via email. To motivate respondents to participate in the survey and ensure data quality, two methods were adopted. First, to encourage the respondents to participate in the survey and increase the response rate, CNY 5 (equivalent to USD 0.7) were rewarded after the questionnaires were checked and approved by the research team. Second, some questionnaires were discarded based on the users’ fill-in time (less than three minutes is assessed to be unqualified) and rules (those with the same answers on all different variables were eliminated; eligible participants had to be at least 18 years of age).

**Figure 3:**
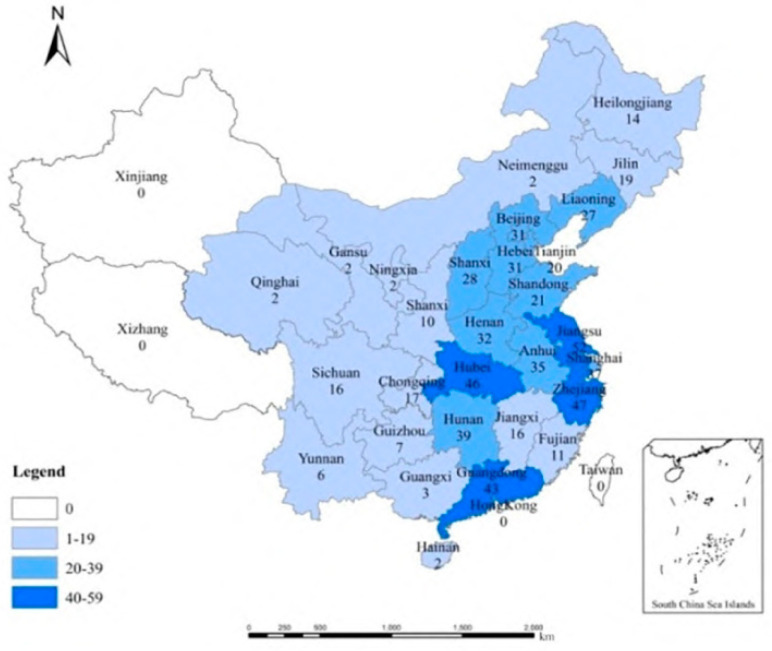
Sample distribution.

Since the *Gongkuai* campaign is a nationwide health campaign, the survey was conducted randomly and was not limited to a specific area. The online survey lasted six weeks (from 2 April to 19 May 2020). In the end, 659 participants completed the questionnaire; 41 questionnaires were deemed invalid by research teams, and 618 valid questionnaires were obtained. The sample distribution is shown in Figure 3, and the detailed profile information of the respondents is presented in Table 1. According to the results, 317 respondents were men, and 301 were women. Approximately 89.6% of respondents were between 20 and 50 years old, and 57.6% had an associate or bachelor’s degree. Almost one-third of the respondents (33.6%) reported that their monthly household income is between CNY 10,000 and CNY 15,000. Overall, 41.6% of those surveyed live in urban areas. In general, the demographic characteristic of the participants, such as gender and residential location, were consistent with the demographic profile of actual Chinese residents. Additionally, age, educational level, and monthly household income were congruent with the demographic profile of Survey Star members, which registered members were relatively young, rich, and well educated [56].

### 3.2. Measures

Each variable was measured using multiple items derived from previous research and modified to fit the research context (see Table 2). Each item was scored on a 5-point scale ranging from strongly disagree (1) to strongly agree (5). Three measurement items for campaign exposure were referenced from the research of Shen et al. [57] and Karletsos et al. [58]. Three items of perceived traditional diet culture were developed based on the research of Zhang et al. [9] and Swierad et al. [5]. According to the studies of Gaspar et al. [59] and Yan et al. [20], three items were developed to measure campaign-related knowledge. Three items of health risk perception were referenced from the work of Lindell and Perry [13] and Yan et al. [20]. Three items of interpersonal communication were referenced from the works of Kim and Grunig [49] and Karletsos et al. [58]. The research teams conducted a pilot survey among several research scholars and commuters, and their feedback and suggestions were used to improve the questionnaire’s quality.

### 3.3. Descriptive Statistics and Correlations

Before testing the research hypotheses, we performed a descriptive statistics analysis to obtain general information about the variables. The results of descriptive statistics and bivariate correlations are presented in Table 3. There are significant associations between each variable among the various constructs; thus, a deeper analysis is warranted. Meanwhile, the square root of the average variance extracted (AVEs) was greater than the correlations between each construct, indicating that the discriminant validity meets the criterion. In addition, it should be noted that some correlations between constructs were higher than the benchmark of 0.6, so a multicollinearity test was needed. The highest variance inflation factor (VIF) found in the analysis was 4.1, indicating that multicollinearity is not a significant problem in this dataset [60].

## 4. Results

Given that the variables were latent, the proposed model was evaluated using the structural equation modeling (SEM) technique. Data analysis was performed in two steps [61]. A confirmatory factor analysis (CFA) was performed to determine whether the questionnaire items accurately measured their intended constructs. After the measurement model was proven to fit well, the second step involved conducting a path analysis to test the hypothesized relationships in the proposed model.

### 4.1. Measurement Model

The measurement model’s fit indicators were listed as follows: χ^2^ = 22.405, df = 80, χ^2^/df = 2.505; TLI = 0.973, CFI = 0.980; RMSEA = 0.049. These figures reveal a good fit between the measurement model and the dataset. Additionally, confirmatory factor analysis (CFA) was implemented to test the construct’s reliability and validity. Cronbach’s alpha value and the composite reliability value were used to evaluate the reliability of constructs. Table 4 shows that Cronbach’s alphas ranged from 0.80 to 0.94, greater than the threshold condition. In addition to this, the composite reliability ranged from 0.84 to 0.94, higher than the benchmark value of 0.7 [62]. Moreover, factor loadings and average variance extracted (AVE) were used to test the convergent validity. The factor loadings ranged between 0.60 and 0.85. The AVEs of all five constructs exceed the criterion of 0.6. According to the above two findings, all constructs have good convergent validity.

### 4.2. Structural Equation Model Analysis

**Figure 4:**
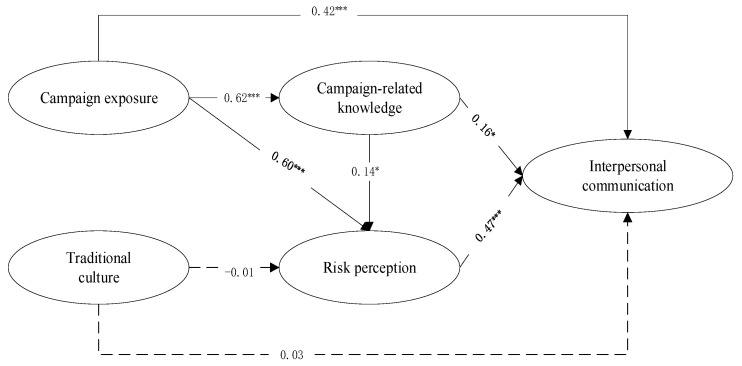
Results of hypothesis testing. Note: * *p* < 0.05 and *** *p* < 0.001.

The structural model’s fit indicators were acceptable as shown in the results (χ^2^ = 150.817, df = 79, χ^2^/df = 1.909; TLI = 0.984, CFI = 0.988; RMSEA = 0.038). t-values (t) and path coefficients (β) are used to test the verified relationships of all constructs in the proposed model, as shown in Figure 4. Campaign exposure significantly influences individuals’ perceived campaign-related knowledge (H1: β = 0.62, t = 12.34) and health-risk perception (H2: β = 0.60, t = 10.42). Individuals who perceive more campaign-related knowledge tend to have a high level of risk perception of not using serving chopsticks (H3: β = 0.14, t = 2.03). However, traditional diet culture fails to significantly predict risk perception (H4 β = −0.01, t = −0.03) and interpersonal communication (H5: β = 0.03, t = 1.00). In addition, individuals who perceive more knowledge about the *Gongkuai* campaign (H6: β = 0.16, t = 2.21) and more risk perception (H7: β = 0.47, t = 9.62) tend to share and discuss information about the *Gongkuai* campaign actively. Finally, exposure to health campaigns positively influences interpersonal communication (H8: β = 0.42, t = 7.71). From these results, we concluded that all but two of the hypotheses (H4 and H5) were supported.

## 5. Discussion and Implication

### 5.1. Discussion

Consistent with previous studies on interpersonal communication in healthcare [11,12], this study demonstrated the positive and significant effect of exposure to health campaigns on interpersonal communication. At the same time, higher levels of campaign exposure also positively improve levels of health awareness and risk perception. In addition, higher levels of campaign-related knowledge are a significant predictor of levels of health risk perception and interpersonal communication, as those who gain more knowledge about the campaign are more likely to perceive the risk of not using serving chopsticks. The previous studies suggest that gaining more knowledge from a mass media campaign in the context of public health crisis influences an individual’s risk perception of unhealthy behaviors positively [18]. Perettiwatel et al. [31], on the other hand, emphasized that if parents do not understand basic human papillomavirus (HPV) and HPV vaccine information, they will not realize the efficacy and benefits of HPV vaccination. Meanwhile, campaign-related knowledge can encourage individuals to share and discuss health campaigns. This finding is consistent with previous studies that show that knowledge about specific health issues [63] and health behaviors [48] lead to more active sharing and discussion of information with others.

Furthermore, the findings indicate that higher levels of health risk perception directly affect individuals’ communication of health campaign messages, implying that improving people’s health risk perception of unhealthy behavior is valuable for motivating interpersonal communication. The finding is consistent with previous studies. For example, Arroyo and Harwood [41] demonstrated that people who perceive a high risk of eating disorders are more likely to discuss obesity actively. Furthermore, Kim and Grunig [49] observed that perception of a problem predicts information behaviors (e.g., information seeking and sharing).

Levels of agreement with traditional diet culture, on the other hand, do not directly predict levels of risk perception. According to a previous study (Zhang et al., 2020), Chinese dining culture has never stopped evolving and is at another critical crossroads as the country itself is undergoing a historic transformation. Extending this idea to the current study, it was seen that, while the media consensus reported that social culture is an impediment to the *Gongkuai* campaign, the changing features of dining culture contribute to the insignificant influence on an individual’s risk perception. Moreover, PADM emphasized that in the context of a risk event, people will be more active in information seeking and processing and will use this information to evaluate the severity of the event to their safety and health [13]. Thus, based on PADM, the current study concluded that the level of agreements with the traditional social culture of sharing utensils would not negatively influence levels of individuals’ perceptions of health risk in the context of a public health crisis (e.g., a devastating pandemic).

Finally, contrary to our expectations, levels of agreement with traditional diet culture did not negatively affect interpersonal communication. One possible reason is that the constantly evolving characteristics of traditional Chinese culture can lead to differentiated interpersonal communication between individuals (active, passive, and neutral). In China, there are two more possible subjective explanations. First, despite the perception that people’s health behaviors are rooted in cultural relationships and interactions [5], their social activities are more powerful and can be explained by their perceived risks from external hazards and warning messages [13]. Second, interpersonal communication can be thought of as reasoned and problem-solving behavior that occurs when a person perceives a problem and its connections [49]. People in China are highly concerned about various aspects of the food chain because of the ongoing pandemic [9]. Meanwhile, the *Gongkuai* campaign has raised public awareness regarding the importance of changing the traditional communal dining style. These feelings of anxiety and awareness encourage people to pay attention to more information about the food chain during COVID-19, which leads to an increase in motivation to engage in communication behaviors.

### 5.2. Implications

This study has a wide range of theoretical implications. First, interpersonal communication is critical for achieving health campaign-targeted outcomes (Jeong & Bae, 2018). Although the *Gongkuai* campaign is vital for improving healthy eating habits, it has received little scholarly attention. The current study considers interpersonal communication as an explicit outcome of campaigns to conduct in-depth research. Meanwhile, the findings can help us better understand the communication processes that underpin the *Gongkuai* campaign’s reactions. Second, using PADM, the current study investigated how health campaign affects individuals’ perceptions of health risk and campaign-related interpersonal communication in the context of post-COVID-19. As a result of this research, the applied range of PADM can be expanded from natural hazards to public health emergencies. Third, this research focuses on the role of traditional diet culture. In contrast to the previous study [15,64], our findings suggest that the influence of traditional social culture on health behaviors is debatable. In the context of a health crisis, the primary factor influencing campaign-generated interpersonal communication is a perception of external health risks.

In addition, two practical implications of this study are proposed. For starters, communal dining is an essential component of Chinese cuisine, distinguished by intimate food sharing at the dinner table. During the COVID-19 epidemic, it is increasingly viewed as a public risk rather than a tradition or traditional diet culture to be treasured in China. However, such a shift is unlikely to occur quickly. Our results revealed that campaigns promoting individuals’ risk perception and health knowledge effectively improve their interpersonal communication. Thus, health campaign designers should take more concrete measures to improve people’s risk perception and health knowledge. Second, a previous study proved that campaigns aimed at changing an old habit are unlikely to have a large impact on outcomes [12]. This study tentatively proposed that an external public crisis serves as a catalyst for this type of health campaign, generating situational motivation to reconsider people’s old behaviors. In the context of an external health crisis, a health campaign aimed at changing old habits is more likely to stimulate interpersonal communication and subsequently improve the campaign’s success. Furthermore, while traditional diet culture is not an obstacle between campaign exposure and interpersonal communication; it is worth noting that if people talk negatively about the *Gongkuai* campaign in a way that reinforces traditional dining culture, new social norms for health behavior may weaken rather than strengthen [42]. Thus, public officials should carefully craft campaign-related information that is mixed with information that reminds people of the health hazard posed by COVID-19 before disseminating it through online websites or newspapers. These efforts could help raise people’s risk perception of not using serving chopsticks and stimulate campaign-related interpersonal communication.

### 5.3. Conclusions

The main goal of *Gongkuai* campaign was to change traditional communal eating habits and reduce public health risks. The current study investigated the determinants of participants’ interpersonal communication about *Gongkuai* campaign using PADM. The results support most hypotheses and provide a better understanding of how health campaigns intrigue interpersonal communication.

## 6. Limitations and Future Directions

There were several limitations to this research. This study focuses on the effects of health campaign exposure, traditional diet culture, perceived knowledge about the *Gongkuai* campaign, and perceived risk of not using serving chopsticks on individuals’ interpersonal communication. Other possible factors such as health literacy and individuals’ attitudes towards health campaigns may affect levels of health risk perception. In addition, given the complexity of communication behavior, many other factors such as conversation partners and situational facilitators may also influence it. Hence, future research should take these factors into account. Second, changes in behaviors—the level at which people used serving chopsticks before and after the *Gongkuai* campaign was not captured because this study was a cross-sectional survey. A longitudinal design should be considered in future research. Finally, the results are limited in generalizability due to the small sample size. Hence, data from more cities and sources should be considered in future studies.

## Figures and Tables

**Table 1:** Profile information of respondents (*n* = 618).

Demographic Characteristic		Frequency	%
Gender	Male	317	51.3
	Female	301	48.7
Age	Less than 20	59	9.6
	20–30	158	25.6
	31–40	188	30.4
	41–50	148	24.0
	More than 50	65	10.4
Education	Senior high school or below	109	17.6
	Associate or bachelor’s degree	318	51.5
	Master’s or higher degree	191	30.9
Monthly Household income	Less than CNY 10,000	133	21.5
	CNY 10,000–15,000	208	33.6
	CNY 15,001–20,000	161	26.1
	More than CNY 20,000	116	18.8
Residential location	Urban	257	41.6
	Suburban	242	39.2
	Rural	119	19.2

**Table 2:** Constructs and measurement items.

Construct	Item	Measurement	Reference
Campaign exposure (CE)	CE1	I often browsed or heard information about *Gongkuai* campaign in the past 2 months	[57,58]
	CE2	I often browse or hear information about *Gongkuai* campaign from traditional sources (e.g., television/radio/newspaper)	
	CE3	I often browse or hear information about *Gongkuai* campaign from social media sites (e.g., Wechat/Weibo/TikTok)	
Traditional diet culture (TDC)	TDC1	I believe the shared use of chopsticks is an important traditional diet culture	[5,9]
	TDC2	Using serving chopsticks is very different from traditional customs.	
	TDC3	I have a duty to uphold the traditional diet culture of dishes being shared communally	
Campaign-related knowledge (CK)	CK1	I know *Gongkuai* campaign policy	[20,59]
	CK2	I have knowledge of how to use serving chopsticks	
	CK3	I know many of the negative aspects of not using serving chopsticks	
Risk perception (RP)	RP1	I worry about the danger of not using serving chopsticks	[13,20]
	RP2	It will be dangerous to dine outside if not using serving chopsticks	
	RP3	Not using utensils will negatively influence my future health	
Interpersonal communication (IC)	IC1	In the past two months, I have had conversation with my family or friends about *Gongkuai* campaign	[49,58]
	IC2	In the past two months, I have had conversations with any relevant person about *Gongkuai* campaign	
	IC3	In the past two months, I have actively looked for chances to share my knowledge and thoughts about *Gongkuai* campaign	

**Table 3:** Discriminant validity and descriptive statistics analysis.

	Mean	Standard Deviation	CE	TDC	CK	RP	IC	$\sqrt{\mathrm{AVE}}$
CE	3.69	0.72	1					0.83
TDC	3.02	1.20	−0.03	1				0.92
CK	3.75	0.77	0.55 **	−0.04	1			0.77
RP	3.63	0.72	0.60 **	−0.04	0.44 **	1		0.81
IC	3.60	0.72	0.67 **	−0.03	0.51 **	0.69 **	1	0.79

Note: ** *p* < 0.01.

**Table 4:** Confirmatory factor analysis results.

Construct	Items	Loadings	Cronbach’s Alpha	Composite Reliability	AVE
Campaign exposure (CE)	CE1	0.876	0.87	0.87	0.69
	CE2	0.837			
	CE3	0.778			
Traditional diet culture (TDC)	MC1	0.855	0.94	0.94	0.85
	MC2	0.971			
	MC3	0.914			
Campaign-related knowledge (CK)	CK1	0.700	0.80	0.81	0.60
	CK2	0.775			
	CK3	0.833			
Risk perception (RP)	RP1	0.822	0.85	0.85	0.65
	RP2	0.809			
	RP3	0.794			
Interpersonal communication (IC)	IC1	0.771	0.83	0.84	0.63
	IC2	0.807			
	IC3	0.804			

## Data Availability

The data set supporting the findings of this study are available in the form of tables and figures in the manuscript file. In case of further information needed it could be obtained from the corresponding author upon reasonable request.
